# 中国年轻骨髓增殖性肿瘤患者的临床和基因突变特征

**DOI:** 10.3760/cma.j.issn.0253-2727.2023.03.004

**Published:** 2023-03

**Authors:** 梦雨 张, 梅 宝, 红霞 石, 晓力 刘, 娜 许, 明辉 段, 俊玲 庄, 新 杜, 玲 秦, 吴函 惠, 蓉 梁, 梅芳 王, 烨 陈, 冬云 李, 威 杨, 古生 唐, 伟华 张, 霞 匡, 伟 苏, 艳秋 韩, 丽梅 陈, 霁虹 许, 卓刚 刘, 健 黄, 春亭 赵, 红艳 佟, 建达 胡, 春燕 陈, 协群 陈, 志坚 肖, 倩 江

**Affiliations:** 1 北京大学人民医院，北京大学血液病研究所，国家血液系统疾病临床医学研究中心，北京 100044 Peking University People's Hospital, Beijing 100044, China; 2 南方医科大学南方医院血液科，广州 510080 Nanfang Hospital, Southern Medical University, Guangzhou 510080, China; 3 中国医学科学院、北京协和医学院北京协和医院血液内科，北京 100730 Peking Union Medical College Hospital, CAMS & PUMC, Beijing 100730, China; 4 深圳市第二人民医院（深圳大学附属第一医院）血液科，深圳 518035 Department of Hematology, Shenzhen Second People's Hospital（First Affiliated Hospital of Shenzhen University）, Shenzhen 518035, China; 5 河南科技大学第一附属医院河南科技大学临床医学院，郑州 471003 The First Affiliated Hospital and College of Clinical Medicine of Henan University of Science and Technology, Zhenzhou 471003, China; 6 首都医科大学宣武医院血液科，北京 100053 Xuanwu Hospital, Capital Medical University, Beijing 100053, China; 7 空军军医大学西京医院血液科，西安 710032 Xi Jing Hospital, The Fourth Military Medical University, Xi'an 710032, China; 8 山西医科大学第二医院血液科，太原 030001 Second Hospital of Shanxi Medical University, Taiyuan 030001, China; 9 首都医科大学附属北京安贞医院血液科，北京 100029 Beijing Anzhen Hospital, Capital Medical University, Beijing 100029, China; 10 北京中医药大学东直门医院血液肿瘤科，北京 100700 Dongzhimen Hospital, Beijing University of Chinese Medicine, Beijing 100700, China; 11 中国医科大学附属盛京医院血液科，沈阳 110020 Shengjing Hospital Affiliated to China Medical University, Shenyang 110020, China; 12 海军军医大学第一附属医院（上海长海医院）血液科，中国人民解放军血液病研究所，上海 200433 Department of Hematology, Institute of Hematology, The First Affiliated Hospital of Naval Medical University, Changhai Hospital, Shanghai 200433, China; 13 山西医科大学第一医院血液科，太原 300012 First Hospital of Shanxi Medical University, Taiyuan 300012, China; 14 开封市中心医院血液科，开封 475000 Kaifeng Central Hospital, Kaifeng 475000, China; 15 北京中医药大学东方医院血液病科，北京 100078 Dongfang Hospital, Beijing University of Chinese Medicine, Beijing 100078, China; 16 内蒙古医科大学附属医院血液内科，呼和浩特 010050 The Affiliated Hospital of Inner Mongolia Medical University, Hohhot 010050, China; 17 西安交通大学第一附属医院血液内科，西安 710061 The First Affiliated Hospital of Xi'an Jiaotong University, Xi'an 710061, China; 18 齐齐哈尔市第一医院血液内科，齐齐哈尔 161005 Department of Hematology, the First Hospital of Qiqihar, Qiqihar 161005, China; 19 浙江大学医学院附属第四医院血液内科，杭州 322000 The Fourth Affiliated Hospital of Zhejiang University School of Medicine, Hangzhou 322000, China; 20 青岛大学附属医院血液科，青岛 266003 The Affiliated Hospital of Qingdao University, Qingdao 266003, China; 21 浙江大学医学院附属第一医院血液科，杭州 310003 The First Affiliated Hospital of College of Medicine, Zhejiang University, Hangzhou 310003, China; 22 福建医科大学附属协和医院血液内科，福州 350001 Fujian Medical University Union Hospital, Fuzhou 350001, China; 23 山东大学齐鲁医院血液科，济南 250012 Shandong University Qilu Hospital, Jinan 250012, China; 24 西北大学医学院，西安 710069 Northwest University School of Medicine, Xi'an 710069, China; 25 中国医学科学院血液病医院（中国医学科学院血液学研究所），实验血液学国家重点实验室，国家血液系统疾病临床医学研究中心，天津 300020 Institute of Hematology and Blood Diseases Hospital, CAMS & PUMC, National Clinical Research Center for Blood Diseases, The State Key Laboratory of Experimental Hematology, Tianjin 300020, China

**Keywords:** 骨髓增殖性肿瘤, 临床表现, 基因突变, NGS分析, Myeloproliferative neoplasms, Clinical manifestations, Genetic mutations, Next-generation sequencing

## Abstract

**目的:**

探究中国年轻骨髓增殖性肿瘤（MPN）患者的临床和基因突变特征。

**方法:**

通过横断面研究，在全国范围内向MPN患者发放调查问卷，根据诊断时年龄分为年轻组（≤40岁）、中年组（41～60岁）和老年组（>60岁），在各疾病类型中比较三组的差异。

**结果:**

共收集到1 727份可供分析的问卷，其中年轻组453例（26.2％），包括原发性血小板增多症（ET）274例、真性红细胞增多症（PV）80例、骨髓纤维化（MF）99例（原发性MF 45例，PV后MF 20例，ET后MF 34例）；男性178例（39.3％），中位年龄31（18～40）岁。与中年、老年MPN受访者相比，年轻MPN受访者中未婚、高学历、无合并症、无合并用药、较低危险度分层占比较高（*P*<0.001）。年轻MPN受访者中以头痛为首发症状患者占比较高（ET：*P*<0.001；PV：*P*＝0.007；MF：*P*＝0.001），脾大的比例在初诊（PV：*P*<0.001）和调研时（ET：*P*＝0.052；PV：*P*＝0.063）最高，而血栓事件的发生率在初诊（ET：*P*<0.001；PV：*P*＝0.011）和调研时（ET：*P*<0.001；PV：*P*＝0.003）均最低。年轻MPN受访者JAK2突变比例最低（ET：*P*<0.001；PV：*P*<0.001；MF：*P*＝0.013），CALR突变比例最高（ET：*P*<0.001；MF：*P*＝0.015），非驱动基因突变（ET：*P*＝0.042；PV：*P*＝0.043；MF：*P*＝0.004）和高分子风险（HMR）突变（ET：*P*＝0.024；PV：*P*＝0.023；MF：*P*＝0.001）的检出率均最低。

**结论:**

与中、老年患者相比，年轻MPN患者有着特有的临床表现和基因突变特征。

## Abstract (en)

**Objective:**

To investigate the clinical and genetic features of young Chinese patients with myeloproliferative neoplasms (MPN).

**Methods:**

In this cross-sectional study, anonymous questionnaires were distributed to patients with MPN patients nationwide. The respondents were divided into 3 groups based on their age at diagnosis: young (≤40 years), middle-aged (41–60 years), and elderly (>60 years). We compared the clinical and genetic characteristics of three groups of MPN patients.

**Results:**

1727 assessable questionnaires were collected. There were 453 (26.2％) young respondents with MPNs, including 274 with essential thrombocythemia (ET), 80 with polycythemia vera (PV), and 99 with myelofibrosis. Among the young group, 178 (39.3％) were male, and the median age was 31 (18–40) years. In comparison to middle-aged and elderly respondents, young respondents with MPN were more likely to present with a higher proportion of unmarried status (all *P*<0.001), a higher education level (all *P*<0.001), less comorbidity(ies), fewer medications (all *P*<0.001), and low-risk stratification (all *P*<0.001). Younger respondents experienced headache (ET, *P*<0.001; PV, *P*＝0.007; MF, *P*＝0.001) at diagnosis, had splenomegaly at diagnosis (PV, *P*<0.001), and survey (ET, *P*＝0.052; PV, *P*＝0.063). Younger respondents had fewer thrombotic events at diagnosis (ET, *P*<0.001; PV, *P*＝0.011) and during the survey (ET, *P*<0.001; PV, *P*＝0.003). JAK2 mutations were found in fewer young people (ET, *P*<0.001; PV, *P*<0.001; MF, *P*＝0.013); however, CALR mutations were found in more young people (ET, *P*<0.001; MF, *P*＝0.015). Furthermore, mutations in non-driver genes (ET, *P*＝0.042; PV, *P*＝0.043; MF, *P*＝0.004) and high-molecular risk mutations (ET, *P*＝0.024; PV, *P*＝0.023; MF, *P*＝0.001) were found in fewer young respondents.

**Conclusion:**

Compared with middle-aged and elderly patients, young patients with MPN had unique clinical and genetic characteristics.

费城染色体阴性骨髓增殖性肿瘤（MPN）是一组以一系或多系骨髓细胞异常增殖的造血干细胞疾病，包括原发性血小板增多症（ET）、真性红细胞增多症（PV）和原发性骨髓纤维化（PMF）[1]。国外文献报道，MPN的中位诊断年龄为60～72岁[2]，年轻患者（≤40岁）占比为8％～12％，年轻患者通常症状负荷、血栓事件发生率较低，生存期较长[3]。我国尚缺乏关于年轻MPN患者临床和基因突变特征的多中心、大样本研究数据资料，因此，我们设计了一项横断面研究，以探究我国年轻MPN患者的临床和基因突变特征。

## 病例与方法

一、研究设计

在2017年9月至2022年3月，向全国范围内的MPN患者发放调查问卷，包括通过互联网发放电子问卷及当面发放纸质版问卷，由经过培训的医生助理协助完成填写。受访者入选标准：①符合2016年WHO骨髓增殖性肿瘤诊断标准[1]，包括ET、PV或MF［包括PMF、PV后MF（Post-PV MF）和ET后MF（Post-ET MF）］[4]–[6]；②诊断年龄≥18岁；③无认知功能障碍。本研究经北京大学人民医院伦理委员会批准，所有受访者均签署知情同意书。

二、调研问卷

问卷分为两部分，第一部分为基础信息，包括：受访者人口学特征（年龄、性别、户籍、婚姻状况、受教育程度），合并症、合并用药，MPN疾病表现（初诊时间、疾病病程、初诊时症状），实验室检查（全血细胞计数、外周血涂片、骨髓涂片、染色体核型、驱动基因突变类型），目前治疗等；第二部分为症状负荷，通过MPN-10量表[7]评估调研时受访者的症状负荷。MPN-10量表包括10个项目：疲劳、早饱感、腹部不适、活动力不佳、注意力不集中、夜间盗汗、皮肤瘙痒、骨痛、发热和体重下降，每个项目分级为0（无）到10（最重），总分0～100分。总分越高，表示症状负荷越重。

三、危险度分层

ET：根据修订版国际血栓预测模型（IPSET-thrombosis）分为极低危（≤60岁、无JAK2突变、无血栓史）、低危（≤60岁、有JAK2突变、无血栓史）、中危（>60岁、无JAK2突变、无血栓史）和高危（有血栓史，或>60岁伴有JAK2突变）[8]。

PV：根据传统的血栓风险模型分为低危（<60岁、无血栓史）和高危（≥60岁/血栓史）[8]。

MF：采用动态国际预后评分系统（DIPSS），根据年龄（>65岁为1分）、WBC（>25×10^9^/L为1分）、HGB（<100 g/L为2分）、外周血原始细胞（≥1％为1分）和体质性症状（有症状为1分）分为低危（0分）、中危-1（1～2分）、中危-2（3～4分）和高危（5～6分）[9]。

四、二代测序（NGS）

非驱动基因突变数据收集自北京大学人民医院302例MPN患者。采集患者外周血样本，使用核酸提取纯化分析仪提取gDNA并构建Illumina标准文库，通过血液肿瘤定制探针进行髓系肿瘤相关基因（包括MPN常见突变基因JAK2、CALR、MPL、CBL、TET2、ASXL1、DNMT3A、IDH1、IDH2、EZH2、U2AF1、SRSF2、SF3B1、TP53、SH2B3等）目标序列捕获，在基因测序仪上进行PE150测序。分析内容包括确定点突变（SNV）、插入和缺失（INDEL）、内部串联重复（ITD）和部分串联重复（PTD）等变异类型。原始数据通过生信分析软件及流程进行比对、分析、注释；为保证变异准确率，对原始变异检测结果进行过滤：每个样本捕获目标区域平均有效深度≥1 000×，支持突变型的reads比对质量和碱基质量值均高于30，并且同时具有正负链支持。

本研究将以下基因突变定义为高分子风险（HMR）突变：①ET患者中SH2B3、SRSF2、IDH2、U2AF1、SF3B1、EZH2和TP53突变[10]–[11]；②PV患者中ASXL1、SRSF2、IDH2突变[10]–[11]；③MF患者中ASXL1、EZH2、SRSF2、U2AF1 Q157、IDH1/2突变[12]–[15]。

五、统计学处理

本调研依据受访患者诊断时年龄分为年轻组（18～40岁）、中年组（41～60岁）、老年组（>60岁），分析三组间社会人口学特征、临床特征和基因突变情况。采用描述性统计分析，连续变量表示以“中位数（范围）”表示，分类变量表示为“数量（比率）”，组间比较时连续变量采用Kruskal-Wallis检验，分类变量采用卡方检验。*P*<0.05被认为差异具有统计学意义。以上统计分析均采用SPSS 22.0软件进行。

## 结果

一、受访者特征

2017年9月至2022年3月，共收集到1 727份可供分析的有效问卷，受访者来自31个省市自治区和直辖市。

1727例受访者的中位年龄52（18～93）岁，男性781例（45.2％）。年轻组453例（26.2％），中位年龄31（18～40）岁，男性178例（39.3％），包括ET 274例、PV 80例、MF 99例（PMF、post-PV MF、post-ET MF分别为45、20、34例）。

在MF受访者中，年轻PMF受访者的占比较低［45.5％（45/99）对62.7％（192/306）对71.0％（115/162），*χ*^2^＝18.249，*P*＝0.001］。与中年、老年MPN受访者相比，年轻MPN受访者未婚、高学历、无合并症、无合并用药、较低危险度分层的比例最高（*P*<0.001）；此外，年轻ET、MF受访者女性比例更高（ET：67.5％对62.8％对56.9％，*χ*^2^＝5.692，*P*＝0.058；MF：60.6％对50.3％对43.2％，*χ*^2^＝7.454，*P*＝0.024）。详见表1～3。

**表1:** 803例原发性血小板增多症患者社会人口学及临床特征

指标	18~40岁	41~60岁	>60岁	*χ*^2^值	*P*值
例数[例/总数（%）]	274/803（34.1）	325/803（40.5）	204/803（25.4）		
年龄[岁，*M*（范围）]	30（18~40）	51（41~60）	68（61~87）	704.101	<0.001
男性[例/总数（%）]	89/274（32.5）	121/325（37.2）	88/204（43.1）	5.692	0.058
城镇户籍[例/总数（%）]	174/273（63.7）	210/322（65.2）	157/202（77.7）	12.173	0.002
婚姻状况[例/总数（%）]				145.997	<0.001
已婚	207/274（75.5）	315/324（97.2）	179/201（89.1）		
未婚	62/274（22.6）	3/324（0.9）	0/201（0.0）		
离异或丧偶	5/274（1.8）	6/324（1.9）	22/201（10.9）		
教育程度[例/总数（%）]				153.497	<0.001
小学	9/273（3.3）	33/315（10.5）	47/199（23.6）		
初中	40/273（14.7）	93/315（29.5）	54/199（27.1）		
高中	33/273（12.1）	100/315（31.7）	38/199（19.1）		
大学及以上	191/273（70.0）	89/315（28.3）	60/199（30.2）		
合并症[例/总数（%）]	99/274（36.1）	204/325（62.8）	165/204（80.9）	100.838	<0.001
合并用药[例/总数（%）]	66/274（24.1）	171/325（52.6）	132/204（64.7）	87.439	<0.001
诊断后治疗[例/总数（%）]	237/271（87.5）	293/323（90.7）	184/202（91.1）	2.260	0.323
病程[年，*M*（范围）]	1.4（0~25）	1.2（0~28）	1.1（0~13.1）	4.524	0.104
脾大[例/总数（%）]	38/140（27.1）	50/242（20.7）	20/132（15.2）	5.920	0.052
血栓史[例/总数（%）]	14/267（5.2）	49/323（15.2）	37/201（18.4）	21.154	<0.001
MPN-10[分，*M*（范围）]	7（0~53）	8（0~69）	11（0~62）	8.039	0.018
异常染色体核型[例/总数（%）]	1/91（1.1）	2/120（1.7）	4/82（4.9）	3.096	0.213
危险度分层[例/总数（%）]				490.445	<0.001
极低危	116/233（49.8）	86/289（29.8）	0/179（0.0）		
低危	106/233（45.5）	151/289（52.2）	0/179（0.0）		
中危	0/233（0.0）	7/289（2.4）	52/179（29.1）		
高危	11/233（4.7）	45/289（15.6）	127/179（70.9）		
目前治疗[例/总数（%）]					
干扰素	104/272（38.2）	103/324（31.8）	41/203（20.2）	17.814	<0.001
羟基脲	52/272（19.1）	123/324（38.0）	121/203（59.6）	81.904	<0.001
阿司匹林	41/272（15.1）	54/324（16.7）	21/203（10.3）	4.122	0.127
其他	30/272（11.0）	27/324（8.3）	7/203（3.4）	9.144	0.010
未治疗	50/272（18.4）	22/324（6.8）	16/203（7.9）	22.998	<0.001

**表2:** 357例真性红细胞增多症患者社会人口学及临床特征

指标	18~40岁	41~60岁	>60岁	*χ*^2^值	*P*值
例数[例/总数（%）]	80/357（22.4）	168/357（47.1）	109/357（30.5）		
年龄[岁，*M*（范围）]	35（20~40）	52（41~60）	67（61~93）	304.927	<0.001
男性[例/总数（%）]	50/80（62.5）	90/168（53.6）	60/109（55.0）	1.814	0.404
城镇户籍[例/总数（%）]	48/77（62.3）	101/164（61.6）	70/103（68.0）	1.187	0.552
婚姻状况[例/总数（%）]				43.283	<0.001
已婚	68/80（85.0）	157/167（94.0）	98/108（90.7）		
未婚	11/80（13.8）	0/167（0.0）	0/108（0.0）		
离异或丧偶	1/80（1.3）	10/167（6.0）	10/108（9.3）		
教育程度[例/总数（%）]				35.137	<0.001
小学	12/80（15.0）	29/166（17.5）	32/105（30.5）		
初中	21/80（26.3）	45/166（27.1）	25/105（23.8）		
高中	7/80（8.8）	54/166（32.5）	22/105（21.0）		
大学及以上	40/80（50.0）	38/166（22.9）	26/105（24.8）		
合并症[例/总数（%）]	46/80（57.5）	126/168（75.0）	99/109（90.8）	28.165	<0.001
合并用药[例/总数（%）]	39/80（48.8）	107/168（63.7）	86/109（78.9）	18.665	<0.001
诊断后治疗[例/总数（%）]	62/77（80.5）	146/162（90.1）	98/106（92.5）	6.956	0.031
病程[年，*M*（范围）]	1.8（0~14.3）	1.4（0~20）	1.0（0~16.8）	1.401	0.496
脾大[例/总数（%）]	31/56（55.4）	61/140（43.6）	30/85（35.3）	5.534	0.063
血栓史[例/总数（%）]	1/78（1.3）	26/166（15.7）	18/108（16.7）	11.949	0.003
MPN-10[分，*M*（范围）]	9（0~75）	9（0~80）	12（0~51）	2.413	0.299
异常染色体核型[例/总数（%）]	1/25（4.0）	1/62（1.6）	0/46（0.0）		0.415
危险度分层[例/总数（%）]					<0.001
低危	77/78（98.7）	119/166（71.7）	0/108（0.0）		
高危	1/78（1.3）	47/166（28.3）	108/108（100.0）		
目前治疗[例/总数（%）]					
干扰素	23/76（30.3）	49/161（30.4）	28/103（27.2）	0.354	0.838
羟基脲	21/76（27.6）	74/161（46.0）	55/103（53.4）	12.199	0.002
阿司匹林	11/76（14.5）	17/161（10.6）	6/103（5.8）	3.741	0.154
其他	17/76（22.4）	17/161（10.6）	15/103（14.6）	5.840	0.054
未治疗	12/76（15.8）	23/161（14.3）	12/103（11.7）	0.684	0.710

**表3:** 567例骨髓纤维化患者社会人口学及临床特征

指标	18~40岁	41~60岁	>60岁	*χ*^2^值	*P*值
例数[例/总数（%）]	99/567（17.5）	306/567（54.0）	162/567（28.6）		
年龄[岁，*M*（范围）]	31（20~40）	52（41~60）	66（61~85）	461.146	<0.001
男性[例/总数（%）]	39/99（39.4）	152/306（49.7）	92/162（56.8）	7.454	0.024
城镇户籍[例/总数（%）]	62/99（62.6）	178/306（58.2）	108/161（67.1）	3.603	0.165
婚姻状况[例/总数（%）]				91.477	<0.001
已婚	75/99（75.8）	294/306（96.1）	145/162（89.5）		
未婚	21/99（21.2）	3/306（1.0）	1/162（0.6）		
离异或丧偶	3/99（3.0）	9/306（2.9）	16/162（9.9）		
教育程度[例/总数（%）]				42.452	<0.001
小学	11/99（11.1）	51/306（16.7）	43/162（26.5）		
初中	14/99（14.1）	90/306（29.4）	51/162（31.5）		
高中	24/99（24.2）	91/306（29.7）	30/162（18.5）		
大学及以上	50/99（50.5）	74/306（24.2）	38/162（23.5）		
合并症[例/总数（%）]	45/99（45.5）	196/306（64.1）	126/162（77.8）	28.252	<0.001
合并用药[例/总数（%）]	36/99（36.4）	171/305（56.1）	104/161（64.6）	20.026	<0.001
诊断后治疗[例/总数（%）]	80/94（85.1）	266/298（89.3）	130/155（83.9）	2.991	0.224
病程[年，*M*（范围）]	2.3（0~38.0）	1.7（0~22.2）	0.9（0~19.3）	21.591	<0.001
脾大[例/总数（%）]	63/73（86.3）	201/255（78.8）	112/144（77.8）	2.412	0.299
血栓史[例/总数（%）]	7/97（7.2）	31/305（10.2）	14/160（8.8）	0.829	0.661
MPN-10[分，*M*（范围）]	14（0~66）	15（0~91）	20（0~62）	3.639	0.162
异常染色体核型[例/总数（%）]	5/33（15.2）	14/113（12.4）	9/72（12.5）	0.185	0.911
危险度分层[例/总数（%）]				45.462	<0.001
低危	5/73（6.8）	8/233（3.4）	4/104（3.8）		
中危-1	53/73（72.6）	141/233（60.5）	36/104（34.6）		
中危-2	12/73（16.4）	77/233（33.0）	47/104（45.2）		
高危	3/73（4.1）	7/233（3.0）	17/104（16.3）		
目前治疗[例/总数（%）]					
芦可替尼	31/97（32.0）	120/295（40.7）	58/157（36.9）	2.472	0.291
干扰素	32/97（33.0）	50/295（16.9）	14/157（8.9）	24.207	<0.001
羟基脲	7/97（7.2）	55/295（18.6）	39/157（24.8）	12.431	0.002
阿司匹林	7/97（7.2）	19/295（6.4）	13/157（8.3）	0.528	0.768
其他	10/97（10.3）	46/295（15.6）	24/157（15.3）	1.727	0.422
未治疗	13/197（13.4）	29/295（9.8）	26/156（16.7）	4.494	0.106

二、临床表现

各年龄组受访者的初诊时症状见表4。年轻MPN受访者有头痛表现的比例最高（ET：23.8％对14.8％对3.9％，*χ*^2^＝35.888，*P*<0.001；PV：35.0％对20.2％对16.5％，*χ*^2^＝9.928，*P*＝0.007；MF：15.3％对13.4％对3.1％，*χ*^2^＝14.109，*P*＝0.001）。年轻ET、PV受访者出现血栓事件的比例最低（ET：4.0％对12.6％对13.8％，*χ*^2^＝16.547，*P*<0.001；PV：1.3％对11.3％对13.8％，*χ*^2^＝8.981，*P*＝0.011）。年轻ET受访者出现乏力（23.1％对32.9％对30.5％，*χ*^2^＝7.32，*P*＝0.026）和皮肤瘙痒（4.4％对6.2％对10.8％，*χ*^2^＝7.985，*P*＝0.018）的比例最低，而发生视物模糊的比例最高（10.6％对8.3％对3.9％，*χ*^2^＝7.146，*P*＝0.028）。年轻PV受访者出现脾大（41.3％对27.4％对11.0％，*χ*^2^＝22.813，*P*<0.001）和手足发绀（21.3％对11.3％对9.2％，*χ*^2^＝6.696，*P*＝0.035）的比例最高。年轻MF受访者出现体重下降的比例最低（16.3％对26.8％对36.6％，*χ*^2^＝12.861，*P*＝0.002）。

**表4:** 1723例受访骨髓增殖性肿瘤（MPN）患者的初诊症状［例（％）］

症状	ET（801例）					PV（357例）					MF（565例）				
	18~40岁（273例）	41~60岁（325例）	>60岁（203例）	*χ*^2^值	*P*值	18~40岁（80例）	41~60岁（168例）	>60岁（109例）	*χ*^2^值	*P*值	18~40岁（98例）	41~60岁（306例）	>60岁（161例）	*χ*^2^值	*P*值
无	134（49.1）	134（41.2）	91（44.8）	3.700	0.157	21（26.3）	57（33.9）	32(29.4）	1.655	0.437	34（34.7）	85（27.8）	48（29.8）	1.712	0.425
脾大	36（13.2）	48（14.8）	19（9.4）	3.303	0.192	33（41.3）	46（27.4）	12（11.0）	22.813	<0.001	41（41.8）	157（51.3）	75（46.6）	2.937	0.230
血象异常	188（68.9）	210（64.6）	135（66.5）	1.203	0.548	56（70.0）	113（67.3）	67（61.5）	1.688	0.430	53（54.1）	177（57.8）	91（56.5）	0.436	0.804
乏力	63（23.1）	107（32.9）	62（30.5）	7.320	0.026	27（33.8）	46（27.4）	30（27.5）	1.206	0.547	39（39.8）	149（48.7）	82（50.9）	3.247	0.197
早饱感	4（1.5）	12（3.7）	6（3.0）	2.800	0.247	1（1.3）	3（1.8）	7（6.4）	5.916	0.052	11（11.2）	52（17.0）	19（11.8）	3.326	0.190
腹部不适	12（4.4）	26（8.0）	15（7.4）	3.382	0.184	9（11.3）	13（7.7）	5（4.6）	2.944	0.230	14（14.3）	96（31.4）	31（19.3）	15.480	<0.001
盗汗	14（5.1）	29（8.9）	13（6.4）	3.430	0.180	13（16.3）	27（16.1）	12（11.0）	1.596	0.450	25（25.5）	75（24.5）	36（22.4）	0.401	0.818
皮肤瘙痒	12（4.4）	20（6.2）	22（10.8）	7.985	0.018	14（17.5）	32（19.6）	24（22.0）	0.603	0.740	13（13.3）	56（18.3）	20（12.4）	3.299	0.192
头痛	65（23.8）	48（14.8）	8（3.9）	35.888	<0.001	28（35.0）	34（20.2）	18（16.5）	9.928	0.007	15（15.3）	41（13.4）	5（3.1）	14.109	0.001
头晕	59（21.6）	88（27.1）	44（21.7）	3.146	0.207	31（38.8）	62（36.9）	35（32.1）	1.037	0.596	24（24.5）	71（23.2）	25（15.5）	4.464	0.107
视物模糊	29（10.6）	27（8.3）	8（3.9）	7.146	0.028	12（15.0）	28（16.7）	17（15.6）	0.128	0.938	9（9.2）	38（12.4）	15（9.3）	1.428	0.490
肢端感觉异常	20（7.3）	35（10.8）	17（8.4）	2.276	0.321	16（20.0）	19（11.3）	10（9.2）	5.392	0.067	5（5.1）	38（12.4）	9（5.6）	8.273	0.016
手足发绀	5（1.8）	12（3.7）	4（2.0）	2.464	0.292	17（21.3）	19（11.3）	10（9.2）	6.696	0.035	5（5.1）	12（3.9）	10（6.2）	1.243	0.537
骨痛	10（3.7）	15（4.6）	8（3.9）	0.363	0.834	6（7.5）	7（4.2）	10（9.2）	2.942	0.230	11（11.2）	30（9.8）	13（8.1）	0.746	0.689
体重下降	14（5.1）	28（8.6）	10（4.9）	4.070	0.131	5（6.3）	14（8.3）	15（13.8）	3.543	0.170	16（16.3）	82（26.8）	59（36.6）	12.861	0.002
血栓栓塞	11（4.0）	41（12.6）	28（13.8）	16.547	<0.001	1（1.3）	19（11.3）	15（13.8）	8.981	0.011	6（6.1）	21（6.9）	9（5.6）	0.299	0.861

注 ET：原发性血小板增多症；PV：真性红细胞增多症；MF：骨髓纤维化

调研时，年轻ET、PV受访者脾大的比例最高（ET：27.1％对20.7％对15.2％，*χ*^2^＝5.920，*P*＝0.052；PV：55.4％对43.6％对35.3％，*χ*^2^＝5.534，*P*＝0.063），而发生血栓事件的比例最低（ET：5.2％对15.2％对18.4％，*χ*^2^＝21.154，*P*<0.001；PV：1.3％对15.7％对16.7％，*χ*^2^＝11.949，*P*＝0.003）。采用MPN-10量表评估MPN受访者的症状负荷，年轻受访者的MPN-10总分均较低，在ET中更为显著（ET：7分对8分对11分，*χ*^2^＝8.039，*P*＝0.018；PV：9分对9分对12分，*χ*^2^＝2.413，*P*＝0.299；MF：14分对15分对20分，*χ*^2^＝3.639，*P*＝0.162）（表1～3）。

三、基因突变

1. 驱动基因突变：可评估驱动基因突变的受访者共1547例，其中ET 708例，PV 317例，MF 522例。在ET受访者中，JAK2、CALR、MPL突变的检出率分别为61.6％（436/708）、14.8％（105/708）、1.6％（11/708），JAK2、CALR、MPL基因突变均阴性（“三阴”）患者占比为22.2％（157/708）；在PV受访者中，JAK2 V617F、JAK2 exon12突变的检出率分别为81.7％（259/317）、4.4％（14/317），无JAK2突变患者占15.5％（49/317）；在MF受访者中，JAK2、CALR、MPL突变的检出率分别为71.6％（374/522）、16.9％（88/522）、2.1％（11/522），“三阴”患者占比为9.4％（49/522）。

进一步比较年轻、中年和老年组的驱动基因突变差异（表5）。年轻ET和MF受访者JAK2突变检出率最低（ET：49.0％对66.7％对70.2％，*χ*^2^＝24.894，*P*<0.001；MF：59.1％对74.1％对74.8％，*χ*^2^＝8.738，*P*＝0.013）、CALR突变检出率最高（ET：23.0％对11.1％对9.9％，*χ*^2^＝19.242，*P*<0.001；MF：26.9％对15.4％对13.3％，*χ*^2^＝8.411，*P*＝0.015），此外年轻ET受访者中“三阴”患者占比最高（ET：27.2％对20.5％对18.2％，*χ*^2^＝5.599，*P*＝0.061）。年轻PV受访者中JAK2 V617F突变检出率最低（60.0％对82.9％对96.9％，*χ*^2^＝38.549，*P*<0.001），JAK2 exon12突变检出率最高（9.3％对4.8％对0.0％，*χ*^2^＝8.780，*P*＝0.012）（表5）。

**表5:** 受访年轻骨髓增殖性肿瘤（MPN）患者的基因突变特征［阳性例数/受检例数（％）］

指标	ET（708例）					PV（317例）					MF（522例）				
	18~40岁	41~60岁	>60岁	*χ*^2^值	*P*值	18~40岁	41~60岁	>60岁	*χ*^2^值	*P*值	18~40岁	41~60岁	>60岁	*χ*^2^值	*P*值
驱动基因突变															
JAK2	117/239（49.0）	192/288（66.7）	127/181（70.2）	24.894	<0.001	-	-	-			55/93（59.1）	212/286（74.1）	107/143（74.8）	8.738	0.013
JAK2 V617F	-	-	-			45/75（60.0）	121/146（82.9）	93/96（96.9）	38.549	<0.001	-	-	-		
JAK2 exon12	-	-	-			7/75（9.3）	7/146（4.8）	0/96（0.0）	8.780	0.012	-	-	-		
CALR	55/239（23.0）	32/288（11.1）	18/181（9.9）	19.242	<0.001	-	-	-			25/93（26.9）	44/286（15.4）	19/143（13.3）	8.411	0.015
MPL	2/239（0.8）	6/288（2.1）	3/181（1.7）	1.344	0.511	-	-	-			1/93（1.1）	5/286（1.7）	5/143（3.5）	1.997	0.368
无突变	65/239（27.2）	59/288（20.5）	33/181（18.2）	5.599	0.061	25/75（33.3）	21/146（14.4）	3/96（3.1）	29.641	<0.001	12/93（12.9）	25/286（8.7）	12/143（8.4）	1.659	0.436
非驱动基因突变	5/46（10.9）	9/45（20.0）	8/22（36.4）	6.183	0.045	3/17（17.6）	6/27（22.2）	6/10（60.0）	6.460	0.040	10/26（38.5）	35/66（53.0）	33/43（76.7）	10.927	0.004
HMR突变	1/46（2.2）	2/45（4.4）	4/22（18.2）	6.957	0.031	0/17（0.0）	2/27（7.4）	3/10（30.0）	6.965	0.031	7/26（26.9）	15/66（22.7）	24/43（55.8）	13.422	0.001

注 ET：原发性血小板增多症；PV：真性红细胞增多症；MF：骨髓纤维化；HMR：高分子风险

2. 非驱动基因突变：可评估非驱动基因突变的受访者共302例（均来自北京大学人民医院），包括年轻组89例，中年组138例，老年组75例。在115例（115/302，38.1％）患者中检出204个突变，其中突变频率较高的前5个依次为ASXL1（48/204，23.5％）、TET2（39/204，19.1％）、DNMT3A（12/204，5.9％）、EZH2（10/204，4.9％）、U2AF1（10/204，4.9％）。检出1个非驱动基因突变者66例（66/115，57.4％），检出2个非驱动基因突变者27例（27/115，23.5％），伴有≥3个非驱动基因突变者22例（22/115，19.1％）。在58例（58/302，19.2％）患者中检出HMR突变，其中检出1个HMR突变者43例（43/58，74.1％），检出2个及以上HMR突变者15例（15/58，25.9％）。

与中、老年受访者相比，年轻MPN受访者具有非驱动基因突变（ET：10.9％对20.0％对36.4％，*χ*^2^＝6.183，*P*＝0.045；PV：17.6％对22.2％对60.0％，*χ*^2^＝6.460，*P*＝0.040；MF：38.5％对53.0％对76.7％，*χ*^2^＝10.927，*P*＝0.004）和HMR突变（ET：2.2％对4.4％对18.2％，*χ*^2^＝6.957，*P*＝0.031；PV：0.0％对7.4％对30.0％，*χ*^2^＝6.965，*P*＝0.031；MF：26.9％对22.7％对55.8％，*χ*^2^＝13.422，*P*＝0.001）的比例均最低（表5）。图1展示了可评估受访者的非驱动基因突变种类和等位基因突变频率（VAF），并比较了各非驱动基因的突变频率在三组之间的差异。其中，ASXL1在年轻ET（2.2％对2.2％对22.7％，*χ*^2^＝12.850，*P*＝0.002）、MF（23.1％对18.2％对44.2％，*χ*^2^＝9.153，*P*＝0.010）受访者中VAF更低；TET2（0.0％对16.7％对30.2％，*χ*^2^＝12.722，*P*＝0.013）和U2AF1（0.0％对3.0％对11.6％，*χ*^2^＝5.676，*P*＝0.059）在年轻MF受访者中VAF更低，而EZH2（15.4％对4.5％对0.0％，*χ*^2^＝7.908，*P*＝0.019）和SH2B3（7.7％对0.0％对2.3％，*χ*^2^＝5.083，*P*＝0.079）在年轻MF受访者中VAF更高。

**图1:**
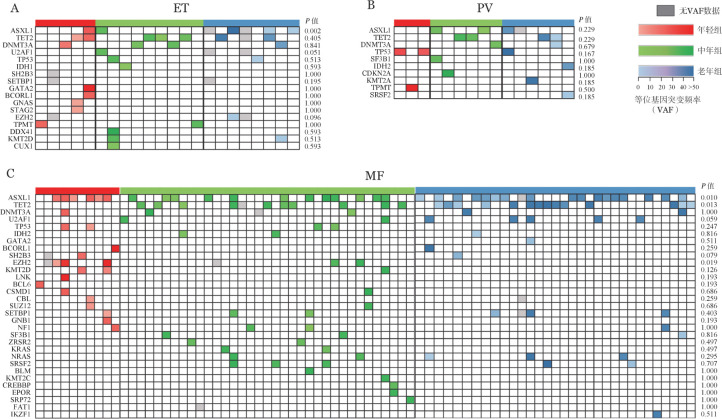
受访骨髓增殖性肿瘤（MPN）患者的非驱动基因突变特征 注 ET：原发性血小板增多症；PV：真性红细胞增多症；MF：骨髓纤维化；HMR：高分子风险

四、治疗

调研时，年轻MPN受访者接受干扰素治疗的比例最高（ET：38.2％对31.8％对20.2％，*χ*^2^＝17.814，*P*<0.001；MF：33.0％对16.9％对8.9％，*χ*^2^＝24.207，*P*<0.001）、羟基脲治疗的比例最低（ET：19.1％对38.0％对59.6％，*χ*^2^＝81.904，*P*<0.001；PV：27.6％对46.0％对53.4％，*χ*^2^＝12.199，*P*＝0.002；MF：7.2％对18.6％对24.8％，*χ*^2^＝12.431，*P*＝0.002），年轻ET受访者未治疗的比例最高（18.4％对6.8％对7.9％，*χ*^2^＝22.998，*P*<0.001）（表1～3）。

## 讨论

本研究是一项针对中国MPN患者的多中心、大样本的横断面研究，旨在评估中国年轻MPN患者的临床和基因突变特征。在本研究中，年轻MPN患者在ET、PV、MF组中的占比分别为34.1％、22.4％、17.5％，远高于西方国家[2]–[3]。

与以往研究结果[3]一致，本研究发现，年轻MPN患者无合并症、无合并用药、较低危险度分层的比例更高；初诊时，年轻MPN患者以头痛为首发症状的比例较高；调研时，年轻MPN患者脾大的发生率较高、血栓事件发生率较低、症状负荷较轻[3],[16]–[17]。此外，本研究也发现，年轻ET、MF患者JAK2突变检出率较低、CALR突变检出率较高；而年轻PV患者JAK2 V617F突变检出率较低，JAK2外显子12突变检出率更高，与以往研究结果[3],[17]相符。以往研究显示，与其他驱动基因突变相比，JAK2突变与高龄、白细胞增多、高血红蛋白水平、低血小板计数及高血栓形成风险相关[18]–[20]；而CALR突变与年龄较低、白细胞计数低、血栓事件发生率低、总生存（OS）期及无白血病生存（LFS）期较长相关[20]–[23]。年轻患者较少具有心血管事件危险因素（高血压、糖尿病、高血脂、动脉粥样硬化等）可解释年轻MPN患者血栓事件发生率低、OS期较长这一现象。

近年来，MPN非驱动基因突变ASXL1、TET2、DNMT3A、EZH2、U2AF1等越来越受到关注。本组病例中，年轻MPN患者非驱动基因突变及HMR突变的检出率均低于中、老年患者；其中，ASXL1在年轻ET和MF患者中突变频率较低，TET2、U2AF1在年轻MF患者中突变频率较低，而EZH2、SH2B3在年轻MF患者中突变频率较高，这是既往文献较少提及的。以往研究已证实，非驱动基因突变尤其是HMR突变是影响MPN患者预后的不利因素[10]–[11],[13]–[15],[24]–[25]；年轻MPN患者较少检出非驱动基因突变以及HMR突变，也可能与良好预后有关。由此可见，非驱动基因突变对于判断MPN患者预后具有重要意义，显示在MPN患者中进行NGS检测是必要的。

本研究有以下局限性：①受访者大多来自大、中型医院，城镇、高学历人口占比偏高，病例选择有所偏倚；②受访者初诊时的临床信息收集不完整；③进行NGS分析的病例数较少；④在MF中未明确区分纤维化前期和纤维化期，未比较非驱动基因突变在二者之间的差异；⑤本研究为横断面研究，未对受访者进行长期随访，未能评估各年龄组MPN患者的并发症（如血栓事件、出血事件、转化为骨髓纤维化或急性白血病）发生率和OS期。

综上所述，与中、老年MPN患者相比，年轻MPN患者症状负荷较轻、血栓事件发生率低、JAK2突变检出率较低、CALR突变检出率较高、非驱动基因突变及HMR突变的检出率均较低。
